# Thrombopenie auf der Intensivstation: Diagnose, Differenzialdiagnose und Therapie

**DOI:** 10.1007/s00740-016-0155-9

**Published:** 2017-01-10

**Authors:** Paul Knöbl

**Affiliations:** Klin. Abt. f. Hämatologie und Hämostaseologie, Medizinische Universität Wien, Währinger Gürtel 18–20, 1090 Wien, Österreich

**Keywords:** Kritische Erkrankung, Hämorrhagie, Thrombozytenerkrankungen, Verbrauchskoagulopathie, Thrombotisch-thrombopenische Purpura, Critical illness, Hemorrhage, Blood platelet disorders, Consumption coagulopathy, Purpura, thrombotic thrombopenic

## Abstract

Thrombozytopenie ist ein häufiges Phänomen in der Intensivmedizin. Eine Vielzahl von Ursachen kann für erniedrigte Plättchenzahlen verantwortlich sein. Da Plättchen Teil der primären Hämostase sind, ist Blutungsneigung die wichtigste Komplikation einer Thrombopenie. Strukturiertes Aufarbeiten der Differenzialdiagnose und Identifikation der Ursache ist essenziell, da die verschiedenen Krankheitsbilder unterschiedliche diagnostische und therapeutische Maßnahmen erfordern. Eine erniedrigte Thrombozytenzahl ist ein starker Prädiktor der Mortalität kritisch kranker Patienten.

Dieser Artikel fasst die Differenzialdiagnose und die diagnostische Aufarbeitung der Thrombopenie in der Intensivmedizin zusammen und gibt einen Überblick über die wichtigsten Krankheitsbilder und die therapeutischen Optionen.

## Abstract (en)

Thrombocytopenia is a frequent phenomenon in intensive care medicine. A variety of conditions can be responsible for low platelet counts. As platelets are part of the primary hemostatic system, bleeding is the most important complication of thrombocytopenia. Proper workup of the differential diagnosis to identify the cause of thrombocytopenia is essential because the various underlying disorders require different diagnostic and therapeutic management strategies. A low platelet count is a strong predictor of outcome in critically ill patients.

This article summarizes the differential diagnosis and diagnostic workup of thrombocytopenia in the critically ill, describes the most important conditions, and gives an overview on therapeutic options and strategies.

Thrombopenie ist bei kritisch kranken Patienten ein häufiges Phänomen, bis zu 60 % können betroffen sein [18]. Etwa die Hälfte dieser Patienten ist schon bei der Aufnahme thrombopenisch, die Übrigen entwickelt die Thrombopenie während des Aufenthaltes auf der Intensivstation. Eine Thrombopenie bei kritisch Kranken ist mit Thrombozytenzahlen <100 G/l definiert, nur <15 % der Patienten haben Werte <50 G/l [18]. Diese Patienten haben auch das höchste Blutungsrisiko. Für invasive Interventionen werden Thrombozytenzahlen >30 G/l angestrebt, bei größeren Eingriffen, je nach Lokalisation auch deutlich höhere Werte.

Es gibt v. a. in der Intensivmedizin eine Vielzahl von Faktoren, die zu einer Reduktion der Thrombozytenzahlen führen können. Die Wichtigsten werden ebenso wie Verfahren zur effizienten Diagnose und Therapie in diesem Artikel beschrieben. Gute Übersichten über Thrombopenie, Gerinnungsstörungen und Blutungsneigung in der Intensivmedizin sind auch hier zu finden: [6, 12, 13, 18].

## Physiologie und Funktion der Thrombozyten

Thrombozyten sind kleine kernlose Zellelemente, die im Blut in einer Zahl von 150–450 G/l zirkulieren. Die Bildung der Thrombozyten erfolgt im Knochenmark durch Abschnürungen des Zytoplasmas von Megakaryozyten. Die Thrombopoese wird durch Thrombopoietin gesteuert, ein Zytokin das in der Leber gebildet wird und über Rezeptoren an den Megakaryozyten ihr Wachstum stimuliert. Die Funktion der Thrombozyten besteht in der primären Hämostase, einem komplexen Prozess, der zur Bildung von Thrombozytenaggregaten an Orten mit Störungen der Endothelintegrität führt. Die Thrombozytenaggregation erfolgt an den A1-Domänen des von-Willebrand-Faktors (VWF), der nach Stimulierung in hochmolekularer Form aus Endothelzellen freigesetzt wird. Durch Scherkräfte im Blutstrom wird er gestreckt, die A1-Domänen werden zugänglich und Thrombozyten binden mit ihren GP-Ib/-IX-Rezeptoren daran. Dadurch werden die Thrombozyten aktiviert und exprimieren GP-IIb/-IIIa-Rezeptoren, an denen Fibrinogen binden und dadurch eine Quervernetzung der Aggregate stattfinden kann.

Auch bei normalen Thrombozytenzahlen kann eine thrombozytäre Blutungsneigung vorhanden sein

Durch die dabei lokalisiert freiwerdenden Phospholipide, Mikropartikel, und Thrombozyteninhaltsstoffe wird die plasmatische Hämostase massiv gefördert. Alle diese Komponenten sind für eine normale Plättchenfunktion notwendig, Defekte in jedem Punkt können Blutungsneigung verursachen. Daher ist die Thrombozytenzahl alleine nicht für eine Blutungsneigung verantwortlich. Auch bei normalen Thrombozenzahlen kann eine thrombozytäre Blutungsneigung vorhanden sein. Im Labor können nur einige dieser Komponenten gemessen werden. Auch die moderne Plättchenfunktionsanalytik, wie PFA100®, Siemens Medical Solutions, Malvern, USA, Multiplate, „fluorescence-activated cell sorting“ (FACS) etc., erfasst nicht alle Aspekte der Plättchenphysiologie. Mehr Details zur Thrombozytenphysiologie finden sich in [3].

## Ursachen und spezifische Therapieansätze

Die Gründe für eine Thrombopenie sind vielfältig. In Tab. 1 sind die Wichtigsten zusammengefasst. Das Erkennen und die richtige zielgerichtete Behandlung erfordern adäquate diagnostische Maßnahmen (Tab. 2) sowie viel klinische Erfahrung in der Interpretation der Testresultate und Auswahl der Therapiestrategie.

**Table 1:** 

*Verminderte Bildung*	Knochenmarkssuppression	Zytostatika, Medikamente, Chemikalien, Alkohol
	Knochenmarksinfiltration	Leukämie, Lymphome, Myelom, myelodysplastisches Syndrom, Infiltration mit anderen Tumorzellen (Knochenmarkskarzinose)
	Aplastische Anämie	
	Vitamin-B-12-Mangel, Folsäuremangel	
	Paroxysmale nächtliche Hämoglobinurie	
	Virusinfekte (Hepatitis C, HIV, EBV, CMV, andere)	
*Vermehrter Verbrauch* *immunologisch*	ITP	
	ITP in der Schwangerschaft	
	Posttransfusionelle Purpura	
	Neonatale Purpura	
	ITP im Kindesalter	
	Medikamenteninduzierte Purpura (Haptene)	Chinin/Chinidin, Sulfonamide, Penizilline, Gold, Rifampicin, u.v.a.m.
	HIT II	
	Antiphospholipidsyndrom	
	Andere Systemerkrankungen (SLE etc.)	
*Vermehrter Verbrauch* *nichtimmunologisch*	Hypersplenismus	Splenomegalie, Leberzirrhose, portale Hypertension, Osteomyelofibrose, Speicherkrankheiten
	DIC	
	Von-Willebrand-Syndrom Typ 2b	
	Mechanische Sequestrierung	Extrakorporale Zirkulation (CVVHF, ECMO, HLM etc.), künstliche Herzklappen, IABP, Pulmonaliskatheter etc.
	Thrombotische Mikroangiopathie	TTP , HUS, HELLP-Syndrom, EPH-Gestose, schwere Vaskulitis, maligne Hypertension
*Sonstige Ursachen*	Hämodilutionsthrombopenie	
	Pseudothrombopenie	
	Präanalytische Fehler, Laborfehler	
	Schwangerschaft	
	Vaskulitis	

*CMV* Zytomegalievirus; *CVVHF* „continuous venovenous hemofiltration“; *DIC* disseminierte intravasale Koagulopathie;* EBV* Ebstein-Barr-Virus, ECMO extrakorporale Membranoxygenierung;* EPH „*edema, proteinuria, hypertension“; *HELLP* „haemolysis, elevated liver enzymes, low platelet count“;* HIT *heparininduzierte Thrombopenie; *HUS* hämolytisch-urämisches Syndrom; *HIV* humanes Immundefizienzvirus;* HLM* Herz-Lungen-Maschine;* IABP* intraaortale Ballonpumpe;* ITP* Autoimmunthrombopenie;* SLE* systemischer Lupus erythematodes; *TTP* thrombotisch-thrombopenische Purpura

**Table 2:** 

Test	Indikation (Verdacht auf)	Bemerkungen
Ausführliche Anamnese	Basisdiagnostik	Mögliche Ursache, Verlauf, Dynamik, Medikamente etc.
Klinische Krankenuntersuchung	Basisdiagnostik	Vor allem Leber, Milz, Lymphknoten, Blutungsneigung, Blutungsmuster, Thrombosen, Organfunktion etc.
Komplettes Blutbild	Basisdiagnostik	
Retikulozyten	Basisdiagnostik	Knochenmarksfunktion, Hämolyse
„Immature platelet fraction“ (IPF)	Basisdiagnostik	Vermehrte/verminderte Bildung
Differenzialblutbild	Basisdiagnostik	Erkennen von Neoplasien
Erythrozytenmorphologie	Basisdiagnostik	Erkennen von Fragmentozyten oder sonstigen Abnormalitäten
Serumchemie, LDH	Basisdiagnostik	
Plasmatische Gerinnung	Basisdiagnostik	Erkennen/Ausschluss von DIC
Thrombozytenzahl aus EDTA-Blut	Pseudothrombopenie	Bei Pseudothrombopenie fast normal
Von-Willebrand-Faktor-Antigen, Ristocetinkofaktor, Multimere	VWS Typ 2b	
Knochenmarkspunktion	Hämatologische Systemerkrankung	
Leukozytentypisierung	Hämatologische Systemerkrankung	Inkl. „PI-linked structures“ (PNH-Ausschluss)
Immunologie (ANA, Cardiolipin-Ak, Thrombozyten-Ak etc.)	Systemerkrankung	
Virologie (Hepatitis C, HIV, EBV, CMV etc.)	Virale Genese	
Blutgruppe, Coombs-Test	Hämolyse	Bei TMA: Coombs-Test negativ
Heparin-PF4-Ak	HIT	Nur bei hohem 4‑T-Score; 80 % falsch positiv
Serotonin-Release-Test	HIT	Nur bei hohem 4‑T-Score; oft falsch positiv
ADAMTS13-Aktivität + Inhibitor	TMA	Beweis/Ausschluss einer TTP
Vitamin B12, Folsäure	Mangel	
Schwangerschaftstest	Basisdiagnostik	Bei möglicher Schwangerschaft

*Ak* Antikörper, *ANA* antinukläre Antikörper, *CMV* Zytomegalievirus, *DIC* disseminierte intravasale Koagulopathie, *EBV* Ebstein-Barr-Virus, *EDTA* Ethylendiamintetraazetat, *DIC* disseminierte intravasale Koagulopathie,* HIT* heparininduzierte Thrombopenie, *HIV* humanes Immundefizienzvirus, *IPF* „immature platelet fraction“, *LDH* Laktatdehydrogenase, *PI* Phosphoinositol, *PNH* paroxysmale nächtliche Hämoglobinurie, *TMA* thrombotische Mikroangiopathie, *TTP* thrombotisch-thrombopenische Purpura, *VWS* von-Willebrand-Syndrom

### Verminderte Thrombozytenbildung

Generell kann eine Bildungsstörung von einem vermehrten Thrombozytenverbrauch unterschieden werden. Da Thrombozyten so wie die Zellen der anderen hämatopoetischen Reihen im Knochenmark gebildet werden, ist bei Knochenmarkserkrankungen die Thrombopenie nie isoliert. Es sind immer auch die Erythozyten- und Leukozytenzahlen vermindert. Reifungsparameter, wie Retikulozytenzahlen und die „immature platelet fraction“ (IFP), sind bei Bildungsstörungen vermindert. Ursachen für eine Knochenmarksdysfunktion sind toxische Knochenmarksschäden (z. B. durch Zytostatika, andere Medikamente, Toxine, Chemikalien, Alkohol, Strahlung), Infektionen, v. a. Virusinfekte, wie humanes Immundefizienzvirus (HIV), Epstein-Barr-Virus (EBV), Zytomegalievirus (CMV), Parvovirus, Immunreaktionen (z. B. aplastische Anämie), Mangelerscheinungen (Vitamin B12, Folsäure) oder andere seltenere Ursachen. Diagnostisch ist bei Verdacht auf eine Bildungsstörung eine Knochenmarksuntersuchung obligat. Die Therapie erfolgt bis zur Behebung der Ursache durch Transfusion von Spenderthrombozyten. Die Transfusionsgrenze zur Verhinderung von Spontanblutungen liegt bei 10 G/l und ist bei Eingriffen oder bei klinisch manifesten Blutungen höher (30–50 G/l).

Eine Thrombopenie aufgrund einer Bildungsstörung kann schon bei Aufnahme auf die Intensivstation vorhanden sein (z. B. nach Zytostatikatherapie). Aber auch während der intensivmedizinischen Behandlung kann eine Knochenmarksdysfunktion auftreten (z. B. als Nebenwirkung mancher Medikamente). Die Arbeit von Rice et al. zeigt eine umfassende Tabelle aller potenziell gefährlichen Medikamente [18], auch bei Priziola et al. gibt es eine gute Übersicht [17].

### Vermehrter Thrombozytenverbrauch

Weitaus häufiger entsteht eine Thrombopenie jedoch durch vermehrten Thrombozytenverbrauch. Die Nachbildung von Thrombozyten im Knochenmark ist langsamer als die Elimination aus dem Blut. Die „immature platelet fraction“ (IPF) ist deutlich erhöht.

#### Immunologische Ursachen

Ein vermehrter Thrombozytenverbrauch kann immunologische Ursachen haben, d. h. antikörpermediierte Prozesse bewirken eine beschleunigte Zerstörung der Thrombozyten (Tab. 1). Antikörper gegen Thrombozytenantigene (z. B. GP IIb/IIIa oder Ib/IX) können spontan oder nach Infektionen als Autoimmunreaktion (Autoimmunthrombopenie*,* ITP), aber auch nach Immunisierung (Transfusionsthrombopenie, Thrombopenie in der Schwangerschaft, haptenmediierte Immunisierung als Medikamentenreaktion [17, 18]) gebildet werden. Diese Formen der Thrombopenie können auch während einer intensivmedizinischen Behandlung auftreten. Vor allem die *Transfusionsthrombopenie* (Antikörperbildung gegen HPA-1a als Reaktion auf Erythrozyten- oder Thrombozytentransfusionen) kann mit sehr niedrigen Thrombozytenzahlen (< 1G/l) und schwerer klinischer Blutungsneigung einhergehen.

Ihre Mortalität ist hoch. Für diese Form der Thrombopenie gibt es kaum wirksame Behandlungsmöglichkeiten. Weitere Thrombozytenkonzentrate, Steroide, hochdosierte Immunglobuline (Ig) oder Thrombozytenkonzentrate sind weitgehend wirkungslos. Die Thrombopenie kann mehrere Wochen anhalten um sich dann spontan zu normalisieren (sofern diese Zeit überlebt wird). Diese Erholungszeit kann mit Thrombopoietinrezeptoragonisten eventuell verkürzt werden. Um das Risiko der Entwicklung einer Transfusionsthrombopenie zu verringern, sollte die Indikation zur Transfusion von Blutprodukten prinzipiell sehr streng gestellt werden (siehe Richtlinien zum „patient blood management“ [4, 19]).

Vor allem die Transfusionsthrombopenie kann mit schwerer klinischer Blutungsneigung einhergehen

Auch eine Behandlung mit Abciximab (ReoPro®, Eli Lilly, Indianapolis, USA), eine Antikörpertherapie, die gegen GPIIb/IIIa gerichtet und beim akuten Koronarsyndrom indiziert ist, kann entsprechend einer artifiziell herbeigeführten ITP eine schwere Thrombopenie verursachen. Diese Thrombopenie ist jedoch transient, klingt nach wenigen Stunden/Tagen wieder ab und kann (und muss) nicht behandelt werden. Steroide sind ebenso wie hochdosierte Immunglobuline, Desmopressin, rekombinanter FVIIa, etc., die zumal (bei zugrunde liegendem akutem Koronarsyndrom) kontraindiziert sind, wirkungslos.

#### Heparininduzierte Thrombopenie

Auch die heparininduzierte Thrombopenie (HIT) ist eine immunologisch mediierte Erkrankung [5, 20]. Die Antikörper sind jedoch nicht gegen Plättchenantigene gerichtet, sondern gegen Komplexe aus Heparin und Plättchenfaktor 4. Diese Antikörper entstehen nach Behandlung von Patienten mit Heparinen (v. a. mit Standardheparin, selten auch mit niedermolekularen Heparinen). Die Antikörperbildung beginnt selten vor dem 5. Tag und fast nie nach dem 10. Tag nach Beginn der Heparintherapie. Die Antigen-Antikörper-Komplexe bewirken eine Fc-mediierte Thrombozytenaktivierung, Mikropartikel- und Tissue-factor-Freisetzung und damit eine arterielle und venöse Thromboseneigung, die massiv und lebensbedrohlich sein kann (Details siehe [5]). Akute-Phase-Reaktionen (Infektionen, Operationen etc.) begünstigen die Entwicklung einer HIT. Die Wahrscheinlichkeit einer HIT nach Behandlung mit niedermolekularen Heparinen ist mit <0,1 % etwa 10-mal geringer als nach Standardheparin (1 %; [1]). Generell wird die Häufigkeit einer HIT weit überschätzt, v. a. kritisch kranke Patienten haben eine Vielzahl von anderen Gründen für eine Thrombopenie.

Die Diagnostik einer HIT ist schwierig. Kritisches Abschätzen der Wahrscheinlichkeit für eine HIT anhand eines etablierten Scoringsystems (4-T-Score; Tab. 3) in Zusammenschau mit der Anamnese, der Medikamenten- und Befundhistorie und dem klinischen Bild ist das wichtigste diagnostische Tool [5]. Es gibt keinen Labortest, der eine HIT beweisen kann. Die häufig verwendeten Tests auf Heparin-PF4-Antikörper sind unspezifisch und in bis zu 80 % der Fälle falsch positiv. Ein Antikörpernachweis ist daher kein Beweis für das Vorliegen einer HIT, nicht einmal bei Patienten mit fallenden Thrombozytenzahlen unter laufender Heparintherapie. Andere Tests, wie z. B. der Serotonin-Release-Test, sind spezifischer, trotzdem aber in vielen Fällen (bis 50 %) falsch positiv. Ein negativer Heparin-PF4-Antikörper-Nachweis schließen eine HIT jedoch mit hoher Wahrscheinlichkeit aus [2, 5, 20].

**Table 3:** 

Punkte	2	1	0
Thrombozytenabfall	um >50 % Nadir ≥20 G/l	um 30–50 % Nadir 10–20 G/l	um <30 % Nadir <10 G/l
Zeitlicher Verlauf (Thrombozytenabfall, Event) – Tage nach Heparinexposition	5–10 Tage (≤1 Tag bei Heparinreexposition innerhalb von <30 Tagen)	>10 Tage oder (<1 Tag bei Heparinreexposition innerhalb von 30–100 Tagen)	<5 Tage
Event auf Heparingabe	Neue Thrombose Hautnekrose an Injektionsstelle Anaphylaktische Reaktion nach i.v.-Bolus	Progressive oder rezidivierende Thrombose oder Hautläsion oder Filterthrombose	Kein Event
Andere Gründe für Thrombozytopenie	Keine	Mögliche	Sichere

^a^Prätestwahrscheinlichkeit für HIT: hoch: 6–8 Punkte; mittel: 4–5 Punkte; niedrig: 0–3 Punkte

Die *Behandlung *einer HIT, so diese gesichert ist (mittlerer + hoher 4‑T-Score, positive Labortests), erfolgt durch Umstellung von Heparin auf ein anderes Antikoagulans in therapeutischer Dosierung (trotz niedrigerer Thrombozytenzahlen). Bewährte alternative für die Behandlung einer HIT zugelassene Antikoagulanzien sind Argatroban (Argatra®, Mitsubishi Tanabe Pharma GmbH, Düsseldorf, Deutschland) und Danaparoid (Orgaran®, Aspen Pharma Ireland Limited, Dublin, Irland).

Akute-Phase-Reaktionen begünstigen die Entwicklung einer heparininduzierten Thrombopenie

Auch Bivalirudin (Angiox®, The Medicines Company, Parsippany, USA), Fondaparinux (Arixtra®, GlaxoSmithKline, Mississauga, Kanada) oder direkte orale Antikoagulanzien, wie Dabigatran (Pradaxa®, Boehringer Ingelheim, Ingelheim, Deutschland), Rivaroxaban (Xarelto®, Bayer HealthCare, Leverkusen, Deutschland), Apixaban (Eliquis®, Bristol-Myers Squibb GmbH & Co. KGaA, München, Deutschland), wurden mit Erfolg verwendet, sind aber (außer bei schon manifester Thromboembolie) nicht explizit für HIT zugelassen.

Vitamin-K-Antagonisten sind in der Akutphase einer HIT verboten, die Gabe von Thromboztenkonzentraten muss vermieden werden [5, 20]! Auch Systemerkrankungen, wie ein systemischer Lupus erythematodes oder ein Antiphospholipidantikörpersyndrom können mit immunologisch bedingter Thrombopenie einhergehen.

#### Nichtimmunologische Ursachen

Häufige Gründe für einen vermehrten Thrombozytenverbrauch sind Erkrankungen von Leber und/oder Milz, z. B. Leberzirrhose, portale Hypertension und Splenomegalie jeglicher Ursache. In der Milz werden Thrombozyten aus der Zirkulation eliminiert. Lebererkrankungen gehen auch oft mit einer systemischen Gerinnungsaktivierung einher, die zur thrombinmediierten Thrombozytenaktivierung führen kann.

##### Mechanische Sequestrierung.

Die moderne intensivmedizinische Therapie umfasst zunehmend mehr Verfahren mit extrakorporaler Zirkulation (chronisch-venovenöse Hämofiltration, verschiedene Dialyseverfahren, extrakorporale kardiopulmonale Ersatz- bzw. Unterstützungsverfahren, intraaortale Ballonpumpen etc.). Dabei kann es an den Schlauchsystemen, Membranen, Oxygenatoren und Pumpen zur mechanischen Sequestrierung von Thrombozyten, aber auch zur Gerinnungsaktivierung und zur thrombinmediierten Thrombozytenaktivierung kommen.

##### Disseminierte intravasale Koagulopathie.

Als disseminierte intravasale Koagulopathie (DIC) bezeichnet man eine systemische Aktivierung der Gerinnung im Rahmen von verschiedenen Erkrankungen (Infektionen, Sepsis, Trauma, Pankreatitis, Malignome, Schwangerschaftskomplikationen, Hämangiome, Toxine, Schlangengifte, systemische Reaktionen; [7, 13]). Die DIC ist durch eine systemische Gerinnungsaktivierung und den Ausfall der regulierenden inhibierenden Mechanismen charakterisiert. Dadurch kommt es zur disseminierten Ablagerung von Fibrinthromben in der Mikro- und Makrozirkulation und in der Folge zur Störungen der Organfunktion bis hin zum Multiorganversagen. Bei Fortschreiten des Prozesses kommt es zum Verbrauch plasmatischer Gerinnungs- und Fibrinolysefaktoren und ihrer Inhibitoren (Verbrauchskoagulopathie). Klinisch stehen initial Thromboseneigung und Mikrozirkulationsstörungen im Vordergrund, bei Verbrauch der Gerinnungsfaktoren und Thrombozyten kommt es dann zu ausgeprägten unstillbaren Blutungen.

Die Maximalform der DIC ist die sog. Purpura fulminans, die z. B. bei Meningokokkeninfektionen vorkommen kann. Dabei bestehen typische landkartenähnliche zentral nekrotische Hautläsionen mit einem hellroten inflammatorischen Randsaum.

Einen entscheidenden *diagnostischen* Hinweis stellt eine progrediente Thrombozytopenie in Kombination mit einem Abfall des Fibrinogens und einem Ansteigen der D‑Dimere dar. In der Routinelaboruntersuchung finden sich darüber hinaus eine verlängerte Prothrombinzeit, eine verlängerte aktivierte partiellen Thromboplastinzeit (aPTT), vermehrt Fibrinabbauprodukte und verminderte Gerinnungsinhibitoren (z. B. Protein C und S, Antithrombin). Fibrinogen wird ebenfalls verbraucht, kann aber als Akutphasenprotein auch scheinbar normal sein. Daher ist der Fibrinogenspiegel immer in Relation zu anderen Akute-Phase-Proteinen (z. B. C‑reaktives Protein) zu beurteilen. Diese Parameter werden in gut evaluierten Scoringsystemen, wie International-Society-on-Thrombosis-and-Haemostasis(ISTH)-DIC-Score, verwendet um eine DIC zu erkennen und den Verlauf zu beurteilen [14]. Diese Scoringsysteme sind jedoch in manchen Situationen nur eingeschränkt beurteilbar (z. B. bei Infektionen, Leberfunktionsstörungen, Hämodilution oder Störungen der Thrombopoese). Daher ist die Beurteilung des Verlaufs (Messungen mehrmals täglich) notwendig, um die Dynamik der Gerinnungsstörung und das Ansprechen auf die Behandlung beurteilen zu können.

Grundlage der *Therapie* einer DIC ist die kausale Therapie der Grunderkrankung. Außerdem muss abgeschätzt werden ob die hämorrhagische oder die thrombogene Komponente überwiegt [10]. Angesichts der Pathophysiologie steht die Regulation der überschießenden Gerinnungsaktivierung im Vordergrund. Das kann durch die Gabe von Heparinen und/oder Inhibitorkonzentraten geschehen. Auch wenn keine guten Studien dazu vorliegen ist eine *Heparinisierung* in der Phase der Thrombosierungen allgemein anerkannt. Aber auch in der Verbrauchsphase kann durch geringe Heparindosen eine Stabilisierung erreicht werden. Standardheparin und niedermolekulare Heparine sind, abhängig von der Patientensituation, mögliche Optionen. *Antithrombinkonzentrate* zeigten in verschiedenen Studien positive Effekte. Vor allem bei der manifesten DIC und sehr niedrigen Antithrombinspiegeln kann die Gabe in Erwägung gezogen werden. Rekombinantes humanes aktiviertes Protein C ist aufgrund des ungünstigen Nutzen-Risiko-Profils nicht mehr verfügbar, jedoch zeigt ein *Protein-C-Zymogen-Konzentrat* (Ceprotin®, Baxter, Unterschleißheim, Deutschland) bei Purpura fulminans ausgezeichnete Ergebnisse [11]. Rekombinantes humanes lösliches *Thrombomodulin* (ART-123) hat sich in einer Studie dem Heparin gegenüber als überlegen erwiesen, ist jedoch nur in Japan verfügbar.

Therapeutisch steht die Regulation der überschießenden Gerinnungsaktivierung im Vordergrund

Bei Patienten mit Blutungen muss eine Substitution prokoagulatorischer Faktoren erfolgen. *Thrombozyten- und Fibrinogenkonzentrate* ermöglichen eine rasche, gut kontrollierte und sichere Gerinnungssubstitution. Bei starker Verminderung der Prothrombinzeit (PTZ) und schweren Blutungen kann die Gabe von *Prothrombinkomplexpräparaten* in Erwägung gezogen werden. Bei lebensbedrohlichen Blutungen (z. B. postpartale Hämorrhagie oder intrazerebrale Blutungen) kann auch *rekombinanter Faktor VII* *a* (NovoSeven®RT, Novo Nordisk, Bagsvaerd, Dänemark) gegeben werden. Die früher oft geäußerte Befürchtung, damit den Prozess anzuheizen, kann heute nicht mehr aufrecht erhalten werden.

Eine Behandlung mit *Blutplasma* ermöglicht eine balancierte Substitution von Gerinnungsfaktoren und -inhibitoren. Um hämostatisch wirksame Effekte zu erzielen, sind jedoch ausreichende Plasmamengen (20–40 ml/kgKG) notwendig, was zur Eiweiß- und Volumenüberladung, transfusionsinduziertem Lungenversagen oder Unverträglichkeitsreaktionen führen kann. Außerdem ist die Bereitstellung durch den notwendigen Auftauprozess langwierig, sodass heute die Plasmatherapie bei DIC nur noch geringen Stellenwert hat.

Die Verabreichung von *Antifibrinolytika,* wie Tranexamsäure (Cyklokapron®, MEDA Pharma GmbH, Bad Homburg v.d. Höhe, Deutschland), sollte nicht durchgeführt werden, da durch die Hemmung der Fibrinolyse die Mikrothrombosen nicht mehr aufgelöst werden und dadurch die Organschädigung verstärkt wird. Nur bei manchen Situationen mit ausgeprägter Hyperfibrinolyse (Promyelozytenleukämie, kavernöse Hämangiome, Hitzschlag oder metastasierende Karzinome), kann durch Antifibrinolytika eine Stabilisierung der Fibrinogenspiegel und eine Reduktion der Blutungen erreicht werden.

##### Thrombotische Mikroangiopathie.

Die thrombotischen Mikroangiopathien (TMA) sind eine Gruppe von Erkrankungen, die zwar einen ähnlichen laborchemischen und klinischen Phänotyp haben, sich aber pathophysiologisch durchaus unterscheiden (Tab. 4; [8]). Gemeinsam ist das Bild einer Coombs-negativen hämolytischen Anämie mit Fragmentozyten, einer Thrombopenie und Zeichen der Organdysfunktion. Diese kann alle Organe betreffen, beeindruckt aber meist als Nierenfunktionseinschränkung, zerebrale Symptomatik (von Kopfschmerzen bis hin zu Ausfällen, Krämpfen oder Insulten), oder Koronarperfusionsstörung (akutes Koronarsyndrom, Infarkt, Rhythmusstörungen, Kardiomyopathie), kann aber auch wie eine akute Pankreatitis, Darmischämie, metabolische Störung, oder „acute respiratory distress syndrome“ (ARDS) imponieren. Oft besteht auch eine ausgeprägte Hypertonie. Dieses Krankheitsbild hat unbehandelt eine Letalität von etwa 90 %.

**Table 4:** 

**TMA mit ADAMTS13-Defizienz = TTP**	
Genetische Ursachen für ADAMTS13-Defizienz	Upshaw-Schulman-Syndrom
Autoantikörpermediierte ADAMTS13-Defizienz = Morbus Moschcowitz	Spontane Autoimmun-TTP
	Sekundäre Autoimmun-TTP (nach Infektionen, Schwangerschaft, Medikamente, Malignomen etc.)
**TMA mit Komplementdysregulation**	
Genetische Ursachen für Komplementüberaktivierung	Familiäres/kongenitales HUS
Autoantikörpermediierte Komplementfaktorblockade = Autoimmuntyp des erworbenen HUS	Spontanes Autoimmun-HUS
	Sekundäres Autoimmun-HUS (nach Infektionen, Schwangerschaft, Medikamente, Malignomen etc.)
**Andere Formen der TMA (einige bezeichnet als atypisches HUS, aHUS)**	
Idiopathisch/spontan	Kein Trigger nachweisbar
Organtransplantation	Nieren, hämatopoetische Stammzellen, Lunge, Herz, Leber etc.
Infektionen	EBV, CMV, HIV etc.
Medikamente	Clopidogrel, Ticlopidin, Cyclosporin, Chinin, Mitomycin C etc.
Malignome	Disseminierte Tumorerkrankungen, Knochenmarkskarzinose
Schwangerschaft	HELLP-Syndrom, Präeklampsie
Toxine	Diarrhöassoziiertes HUS: *Escherichia* * c* *oli, Shigella* etc.

*CMV* Cytomegalievirus, *EBV* Ebstein-Barr-Virus, *HELLP* „haemolysis, elevated liver enzymes, low platelet count“,* HIV* humanes Immundefizienzvirus, *HUS* hämolytisch-urämisches Syndrom, *TMA* thrombotische Mikroangiopathie, *TTP* thrombotisch-thrombopenische Purpura, *VWS* von-Willebrand-Syndrom

Das *diagnostische* Panel ist, analog zur generellen Abklärung einer Thrombopenie, darauf ausgelegt, alle möglichen Ursachen zu evaluieren. Beweisend für eine thrombotisch-thrombopenische Purpura (TTP) ist ein schwerer ADAMTS13-Mangel. Für die anderen Formen der TMA gibt es keine beweisenden Tests. Wichtig ist jedoch auch ein enges Monitoring der Organfunktionen (Kreatinin, Kreatinphosphokinase, Troponin, Elektrokardiogramm, Elektroenzephalogramm, kraniale Computertomographie etc.), da die Organdysfunktion lebensbedrohlich werden kann.

Generell erfordert die *Behandlung* einer akuten Episode einer TMA viel Erfahrung und ein koordiniertes Zusammenspiel aller beteiligten Abteilungen [9]. In den meisten Fällen ist eine intensivmedizinische Betreuung notwendig. Vor allem zerebrale Minderperfusion (bis hin zu ischämischen Insulten) und der Verschluss von Koronararterien führen zum Tod des Patienten. Daher hat die Verbesserung der Organperfusion in der Frühphase hohe Priorität. Die Unterbrechung des Autoimmunprozesses ist jedoch die kausale Therapie.

*Plasmaaustausch:* Die Einführung der Plasmaaustauschtherapie in den 1970er-Jahren hat zu einer dramatischen Verbesserung der Mortalität geführt, diese liegt aber immer noch bei etwa 20 %. Dabei wird täglich das 1‑fache Plasmavolumen (etwa 50 ml/kgKG) entfernt und mit Spenderplasma („fresh frozen plasma“, FFP, Octaplas®, Octapharma®, Langenfeld, Deutschland) ersetzt. Dadurch werden ultragroße von-Willebrand-Faktor-Multimere, Autoantikörper, Hämolyseendprodukte und Thrombozytenaggregate aus der Zirkulation entfernt und ADAMTS13 und normaler von-Willebrand-Faktor zugeführt. Diese Therapie wird ohne Unterbrechung durchgeführt, bis sich Thrombozytenzahl, Laktatdehydrogenase und Organfunktionen normalisiert haben. Bei schlecht respondierenden Patienten kann die Intensität auf das 1,5-fache Plasmavolumen bzw. 2‑mal täglich auf das 1‑fache Plasmavolumen gesteigert werden. Wenn diese Therapiemöglichkeit nicht besteht, sind 20 ml/kgKG Plasma zu infundieren und der Patient umgehend in ein spezialisiertes Zentrum zu transferieren. Bei bekanntem kongenitalem ADAMTS13-Mangel ist es ausreichend, 3–4 Beutel Plasma zu infundieren.

*Kortikosteroide:* Der Autoimmunprozess, der zur erworbenen TTP führt, kann mit Steroiden (z. B. 1,5 mg/kgKG Prednisolon täglich) behandelt werden. Dies ist heute Teil des Standardbehandlungsprotokolls.

*Aggregationshemmer:* Im Fall schwerer Mikrozirkulationsstörungen (v. a. zerebral und koronar) besteht trotz niedriger Thrombozytenzahlen eine Indikation für Plättchenaggregationshemmer. Aufgrund der Hemmung der durch Adenosindiphosphat (ADP) induzierten Plättchenaggregation (ADP wird bei Hämolyse freigesetzt) scheint Clopidogrel (75 mg täglich, bei Thrombopenie ohne Aufsättigung) sinnvoller zu sein als Azetylsalizylsäure. Klinische Studien hierzu gibt es jedoch nicht. TTP-Patienten versterben jedoch nicht an Blutungen, sondern am Myokardinfarkt oder am Insult.

*Splenektomie:* Bei primär schlecht respondierenden oder häufig relapsierenden Patienten ist die Splenektomie (nach entsprechender Vakzinierung) eine gute Möglichkeit, dauerhafte Remissionen zu erreichen.

*Rituximab:* Dieser Anti-CD20-Antikörper ist zur Behandlung von malignen Lymphomen und von Autoimmunerkrankungen zugelassen. Er zerstört immunglobulinbildende Lymphozyten und unterbricht damit die Nachbildung der Anti-ADAMTS13-Antikörper.

*Andere Immunsuppressiva:* Cyclophosphamid, Azathioprin, Cyclosporin A, Vincristin etc. wurden bisher mit eher mäßigem Erfolg zur Behandlung der TTP eingesetzt. Sie sollten daher nicht Therapie der ersten Wahl sein und nur in speziellen Fällen verwendet werden.

*ALX0081 (Caplacizumab; Ablynx NV, Zwijnaarde, Belgien):* Dieses „nanobody“ (variable Domäne eines Immunglobulins) blockiert die A1-Domänen des von-Willebrand-Faktors und damit die Andockstelle der thrombozytären GP-Ib/-IX-Rezeptoren. Die dadurch erzielte Verhinderung der Plättchenaggregation sollte daher zu einer raschen Verbesserung der Organfunktionen führen. Zurzeit läuft eine Phase-II-Studie an Patienten mit akuten Schüben einer TTP.

*ARC1779:* Dieses Aptamer (RNA/DNA-Fragment) blockiert ebenfalls die A1-Domänen des von-Willebrand-Faktors. Nachdem die Autoren vor 4 Jahren einen Patienten mit refraktärer TTP auf Compassionate-use-Basis mit dieser Substanz behandelt hatten, führten sie weitere Studien bei Patienten mit akuten Schüben einer TTP durch. Dabei konnte ein signifikanter Anstieg der Thrombozytenzahlen nachgewiesen werden. Diese Substanz wird jedoch von der Herstellerfirma nicht weiterentwickelt.

*Immunadsorption:* Durch Adsorption von Immunglobulinen an Staphylokokkenprotein A oder Anti-human-Immunglobulin-Säulen können Immunglobuline aus der Zirkulation entfernt werden. Dieses komplizierte und teure Verfahren soll jedoch nur eingesetzt werden, wenn das Ansprechen auf die konventionelle Therapie nicht ausreichend ist.

*Rekombinantes humanes ADAMTS13:* Das rekombinante Enzym wird zurzeit in klinischen Studien bei angeborenem und erworbenem ADAMTS13-Mangel getestet. Vielversprechende Resultate werden erwartet.

*Eculizumab *(Soliris®, Alexion Pharmaceuticals, Zürich, Schweiz)*:* Die Blockade des Komplementsystems hat bei TMA-Formen mit Komplementüberaktivierung gute Erfolge gezeigt. Die Substanz ist für das aHUS sogar zugelassen.

*Supportive Therapie:* Die Substitution von Erythrozytenkonzentraten bei schwere Anämie, intensivmedizinische Überwachung, gegebenenfalls Sedierung und Beatmung, Nierenersatztherapie etc. stellen wesentliche Therapiebestandteile dar. Die Gabe von Thrombozytenkonzentraten ist, auch bei sehr niedrigen Werten, zumindest in der Frühphase der Behandlung *kontraindiziert*.

### Verschiedene andere Ursachen für eine Thrombopenie

Intensivmedizinische Maßnahmen gehen oft mit einer *Hämodilution* einher (z. B. Sepsistherapie, Behandlung von Kreislaufversagen, Massivblutungen etc., aber auch Flüssigkeitsretention bei Nierenfunktionseinschränkung oder Hormon- und Elektrolytstörungen). Dabei werden alle Blutbestandteile, darunter auch die Thrombozyten, verdünnt.

Eine *Pseudothrombopenie* entsteht in vitro durch Thrombozytenaggregation im mit Ethylendiamintetraazetat (EDTA) antikoaguliertem Blut. Die Bestimmung der Thrombozytenzahlen aus Zitratblut kann helfen, diese klinisch irrelevante präanalytische Störung zu diagnostizieren.

Weitere *präanalystische Fehler* (Abnahmefehler, Fehler beim Probentransport und der Probenaufarbeitung etc.) können eine Thrombopenie vortäuschen.

## Allgemeine therapeutische Optionen

Generell soll ohne Abklärung der Ursache der Thrombopenie keine Behandlung erfolgen. Das unkritische Verabreichen von Thrombozytenkonzentraten bei Thrombopenie stellt einen Kunstfehler dar. Vor jeder therapeutischen Intervention muss festgelegt werden, warum diese notwendig ist. Das Blutungsrisiko muss gegen das Risiko möglicher Komplikationen einer Behandlung abgewogen werden. Details zu spezifischen Therapien sind in den entsprechenden Abschnitten angegeben (Tab. 5).

**Table 5:** 

	Dosis	Indikation	Kontraindikation (*KI*)
*Thrombozytenkonzentrate*	1–2/Tag	Bildungsstörung, Hämodilution, DIC, präinterventionell	Absolute KI: TTP, TMA, HIT Relative KI: ITP
*Thrombopoietinrezeptoragonisten* *Romiplostin* *Eltrombopag*	1–9 µg/kgKG und Woche s.c. 50–75 mg/Tag p.o.	ITP, Bildungsstörung	Relative KI: alle Off-label-Anwendungen
*Kortikosteroide*	1 mg/kgKG und Tag	ITP, TTP	
*Immunglobuline*	1 g/kgKG i.v. Tag1 und 2	ITP und lebensbedrohliche Blutungen	
*Immunsuppression, Rituximab*		ITP, TTP	
*Splenektomie*		ITP, TTP, TMA	
*Desmopressin*	0,4 µg/kgKG, KI direkt vor Thrombozytenkonzentrat	Refraktäre Thrombopenie und lebensbedrohliche Blutungen	Nierenversagen, akutes Koronarsyndrom
*Rekombinanter Faktor VIIa*	90 µg/kgKG i.v. (alle 2 h)	Ultima Ratio bei lebensbedrohlichen Blutungen	

*DIC* disseminierte intravaskuläre Koagulopathie, *HIT* heparininduzierte Thrombopenie, *ITP* Autoimmunthrombopenie, *TMA* thrombotische Mikroangiopathie, *TTP* thrombotisch-thrombopenische Purpura

### Thrombozytenkonzentrate

Die Transfusion von Thrombozytenkonzentraten ist nur bei manifester Blutungsneigung aus thrombozytärer Ursache indiziert. Eine prophylaktische Gabe zur Verhinderung von Blutungen wird kontrovers diskutiert, ist jedoch in bestimmten Situationen sicher notwendig (rezente Blutungen, präoperativ etc.). Es existiert jedoch kein anerkannter Grenzwert, unter dem substituiert werden muss. Sicherlich kontraindiziert bzw. wirkungslos sind Thrombozytenkonzentrate bei TTP, TMA, HIT und ITP.

Unerwünschte Wirkungen von Thrombozytenkonzentraten sind Immunisierung (Transfusionsthrombopenie), Refraktärität, allergische Reaktionen, transfusionsassoziiertes Lungenversagen, Zitrattoxizität und Übertragung von Infektionen. Eine Übersicht dazu findet sich in [15].

### Thrombopoietinrezeptoragonisten

Seit einigen Jahren sind 2 Substanzen zur Behandlung der Autoimmunthrombozytopenie zugelassen, die über Stimulation von Thrombopoietinrezeptoren auf Megakaryozyten die Thrombopoese stimulieren: die Thrombopoietinrezeptoragonisten (TPRA) Romiplostim (Nplate®, Amgen, München, Deutschland) und Eltrombopag (Revolade®, GlaxoSmithKline, Mississauga, Kanada; [16]). Beide bewirken innerhalb von 3 Wochen einen deutlichen Anstieg der Thrombozytenzahlen bei den meisten Patienten. Romiplostim wird 1‑mal pro Woche subkutan verabreicht (Dosis 1–9 µg/kgKG), Eltrombopag täglich oral gegeben (Dosis 25–75 mg/Tag). Auch wenn dazu klinische Studien noch fehlen können diese Substanzen auch bei manchen anderen Formen der Thrombopenie wirksam sein.

### Steroide

Bei immunologisch mediierten Thrombopenien (ITP, TTP, HIT etc.) können Steroide durch ihren immunsuppressiven Effekt die Nachbildung der Antikörper reduzieren. Sie sind daher ein fester Bestandteil der Behandlung und werden üblicherweise in einer Dosis von 1 mg/kgKG begonnen.

### Hochdosierte Immunglobulininfusionen

Die am schnellsten wirksame Behandlung einer Autoimmunthrombopenie ist die hochdosierte Gabe von IgG-Konzentraten (Dosis: je 1 g/kgKG an Tag 1 und 2). Die Wirkung setzt nach 1–2 Tagen ein und verschwindet nach 1–2 Wochen wieder. Diese Therapie ist teuer und nur als Notfallbehandlung bei lebensbedrohlichen Blutungen sinnvoll.

### Desmopressin

Desmopressin erhöht nicht die Anzahl der Thrombozyten, kann aber die Wirkung der vorhandenen Plättchen verbessert. Desmopressin wird 1‑mal täglich als Kurzinfusion gegeben (0,4 µg/kgKG, maximal 4 Tage) und bewirkt eine Ausschüttung von von-Willebrand-Faktor aus den Endothelzellen.

### Rekombinanter Faktor VIIa

Als Ultima Ratio bei vital bedrohlichen Blutungen kann auch versucht werden, mit rekombinantem Faktor VIIa (Dosis: 90 µg/kgKG) eine Blutstillung zu erreichen. Die Substanz ist bei Thrombopenie zwar weniger, aber doch auch wirksam.

## Fazit für die Praxis

- Eine Thrombopenie ist häufig bei kritisch kranken Patienten und kann eine Vielzahl von Ursachen haben.
- Nur ein strukturiertes Aufarbeiten der möglichen Differenzialdiagnosen ermöglicht eine sinnvolle Behandlung.
- Die Konsultation von erfahrenen Hämatologen/Hämostaseologen ist dabei oft hilfreich.
- Das unbedingte Erzielen von gewissen Thrombozytengrenzwerten ist nicht durch klinische Studien belegt.
- Die unkritische Substitution von Thrombozytenkonzentraten kann lebensbedrohliche Nebeneffekte haben.
